# Hypercholesterolemia promotes autoantibody production and a lupus‐like pathology via decreased DNase‐mediated clearance of DNA

**DOI:** 10.1111/jcmm.17556

**Published:** 2022-09-13

**Authors:** Umesh Kumar Dhawan, Andreas Margraf, Maciej Lech, Manikandan Subramanian

**Affiliations:** ^1^ Centre for Biochemical Pharmacology, William Harvey Research Institute, Barts and The London School of Medicine and Dentistry Queen Mary University of London London UK; ^2^ LMU Hospital Department of Medicine Munich Germany; ^3^ CSIR‐Institute of Genomics and Integrative Biology New Delhi India; ^4^ Academy of Scientific and Innovative Research Ghaziabad India

**Keywords:** autoantibody, autoimmune, cholesterol, DNase, extracellular DNA

## Abstract

Hypercholesterolemia exacerbates autoimmune response and accelerates the progression of several autoimmune disorders, but the mechanistic basis is not well understood. We recently demonstrated that hypercholesterolemia is associated with increased serum extracellular DNA levels secondary to a defect in DNase‐mediated clearance of DNA. In this study, we tested whether the impaired DNase response plays a causal role in enhancing anti‐nuclear antibody levels and renal immune complex deposition in an *Apoe* ^*−/−*^ mouse model of hypercholesterolemia. We demonstrate that hypercholesterolemic mice have enhanced anti‐ds‐DNA and anti‐nucleosome antibody levels which is associated with increased immune complex deposition in the renal glomerulus. Importantly, treatment with DNase1 led to a decrease in both the autoantibody levels as well as renal pathology. Additionally, we show that humans with hypercholesterolemia have decreased systemic DNase activity and increased anti‐nuclear antibodies. In this context, our data suggest that recombinant DNase1 may be an attractive therapeutic strategy to lower autoimmune response and disease progression in patients with autoimmune disorders associated with concomitant hypercholesterolemia.

## 1 INTRODUCTION

Extracellular DNA released from dying cells is a potent damage‐associated molecular pattern that can stimulate immune cells and exacerbate inflammation.^1^ Consequently, the persistence of high levels of extracellular DNA is positively correlated with disease severity and poor recovery in several autoimmune and inflammatory diseases.^2^, ^3^, ^4^, ^5^ Additionally, the persistence of extracellular DNA likely triggers the activation and production of antinuclear antibodies including anti‐ds‐DNA and anti‐nucleosome antibodies which are pathogenic in several autoimmune disorders.^6^ In this context, extracellular DNA is both a trigger and a potential biomarker for autoimmune rheumatic diseases.^7^

The levels of extracellular DNA in circulation are tightly regulated and maintained at a low level by two endonucleases, DNase1 and DNase1L3, which are present in serum and other biological fluids.^8^, ^9^ The basal levels of DNases are sufficient to maintain extracellular DNA levels under homeostatic conditions. However, acute elevation of extracellular DNA, such as that happens during an acute inflammatory response, requires rapid systemic elevation of DNase activity to restore homeostasis and prevent the onset of an inflammatory response.^10^ Interestingly, several autoimmune diseases are associated with decreased systemic DNase activity, increased extracellular DNA levels, and increased anti‐ds‐DNA autoantibodies.^10^, ^11^, ^12^, ^13^, ^14^ For example, the decreased DNase activity in systemic lupus erythematosus (SLE) is associated with either single nucleotide polymorphisms (SNPs) or mutations in DNase1/DNase1L3^15^, ^16^, ^17^, ^18^, ^19^, ^20^ or the presence of neutralizing antibodies which block the activity of DNase1/DNase1L3.^21^, ^22^ Similarly, the loss of DNase1 or DNase1L3 in mice is associated with a lupus‐like pathology.^23^, ^24^, ^25^

Lupus and other autoimmune diseases are associated with hypercholesterolemia and hypertriglyceridemia which affects about 36%–60% of patients.^26^ Dyslipidaemia in lupus patients is presumably triggered by autoantibodies against lipoprotein lipase^27^ and by high levels of inflammatory cytokines such as TNF‐α^28^ MCP‐1 and IL‐6.^29^ In turn, dyslipidaemia accelerates the progression of atherosclerotic cardiovascular disease which accounts for significant morbidity and mortality in patients with several autoimmune disorders including SLE and RA.^30^, ^31^, ^32^, ^33^

Interestingly, dyslipidaemia also accelerates autoimmune responses as demonstrated by the elevated systemic inflammation and increased titres of autoantibodies against ds‐DNA and nucleosome and a robust autoimmune lupus‐like phenotype in hypercholesterolemic *Apoe* ^*−/−*^ and *Ldlr* ^*−/−*^ mice.^14^, ^34^, ^35^, ^36^ Similarly, hypercholesterolemic individuals have increased levels of autoantibodies against ds‐DNA.^14^ Mechanistically, dyslipidaemia is known to enhance B‐cell mediated auto‐antibody production and promote follicular helper T cell (TFH) expansion in an IL‐27‐dependent manner.^14^ Additionally, hyperlipidaemia is known to enhance immune complex‐mediated complement activation and the development of nephritis in a model of Sle16‐congenic mice on a *Ldlr* ^*−/−*^ background.^37^ Recently, we demonstrated that humans with hypercholesterolemia as well as hypercholesterolemic mice have increased circulating ds‐DNA levels consequent to a defect in release of DNase resulting in heightened inflammation and accelerated atherosclerosis.^10^ However, whether the defective release of DNase and the consequent persistence of ds‐DNA triggers anti‐ds‐DNA antibody production during hypercholesterolemia is not known.

In this study, we show that hypercholesterolemic humans and mice have increased levels of anti‐ds‐DNA and anti‐nucleosome autoantibodies. Additionally, hypercholesterolemic mice show increased immune complex deposition in the renal glomeruli suggesting that these autoantibodies promote the development of immune‐mediated pathological consequences. Most importantly, we demonstrate that the increased levels of autoantibodies and the immune complex deposition in the kidney can be ameliorated by exogenous supplementation of DNase1 in hypercholesterolemic mice. These data highlight that reversal of the defective DNase response during hypercholesterolemia may be a novel therapeutic strategy to decrease autoantibody production and slow the progression of autoimmune pathology.

## 2 MATERIALS AND METHODS

### 2.1 Animal model and maintenance

*Apoe* ^*−/−*^ mice on a *C57BL/6J* genetic background were maintained at the animal facility of CSIR‐Institute of Genomics and Integrative Biology, India. The mice were exposed to a 12 h light–dark cycle and were housed in a temperature‐ and humidity‐controlled room in individually ventilated cages. To induce moderate and severe hypercholesterolemia, 8–10‐week‐old female *Apoe* ^*−/−*^ mice were fed either chow diet or western‐type diet (D12079B, Research Diets, 40% Kcal from fat, 0.2% cholesterol), respectively, for 16 weeks. Appropriate groups of mice received DNase1 injections (400 U/mice intraperitoneal) three times a week for 4 weeks. All animal experiments were conducted with the approval of the Institutional Animal Ethics Committee of CSIR‐Institute of Genomics and Integrative Biology, New Delhi, India.

### 2.2 Isolation of plasma and serum

Blood from mice was collected from either the lateral tail vein or by cardiac puncture in mice undergoing terminal procedures. Blood was collected into either ETDA‐coated microtubes (Sarstedt Catalogue number: 41.1395.005) for plasma and non‐heparinized microtube for isolation of serum. The samples were centrifuged for 10 min at 450 *g* and the serum and plasma were collected from the supernatant. Human blood was collected after obtaining informed consent from apparently healthy adult volunteers with no history of metabolic, endocrine, or autoimmune disorders. The blood was transferred into an EDTA‐coated tube. The isolated plasma and serum were transferred to a fresh tube and stored at −80°C until use. Human studies were conducted with the approval of the Institutional Human Ethics Committee of CSIR‐Institute of Genomics and Integrative Biology, New Delhi, India.

### 2.3 Measurement of extracellular ds‐DNA

Extracellular ds‐DNA concentration in mouse serum and human plasma was measured using Pico green assay kit (Catalogue number: P7589) as per manufacturers' protocol. Briefly, DNA standards were prepared using calf thymus DNA. Serum samples at 1:50 ratio with standards were loaded in 96‐well plates in 100 μl volume. An equal volume of Quant‐iT™ PicoGreen® reagent was added and incubated at room temperature for 5 min before measuring fluorescence at 480/520 nm in NOVOstar fluorimeter (BMG LABTECH).

### 2.4 Quantification of total DNase activity by single radial enzyme diffusion (SRED) assay

SRED assay was conducted for measuring total DNase activity in mouse serum as described previously.^10^ Briefly, 55 μg/ml of salmon testes DNA (Cat # D1626, Sigma) was resuspended in a buffer containing Mn^2+^ (20 mM Tris–HCl pH 7.8, 10 mM MnCl2, 2 mM CaCl_2_, and 2 μl SYBR safe). DNA‐containing solution was heated at 50°C for 10 min and mixed with an equal volume of 2% agarose (Sigma‐Aldrich). The mixture was poured onto a plastic casting tray and left at room temperature till it solidified. Wells of ~0.1 mm diameter were created using a 20 μl tip. 2 μl of murine serum or DNase1 Standard (AMPD1‐1KT, Sigma‐Aldrich) were loaded into wells and gels were incubated for 6 h at 37°C in a humidified chamber followed by image acquisition using UV excitation in a Gel Doc system (FluorChem E system). The area of DNA clearance appears as a dark band which is analysed using ImageJ. The DNase activity in the sample was calculated by interpolation of data obtained from DNase1 standards of known unit activity. 1 unit (U) of DNase1 completely digests 1 μg of DNA to oligonucleotides in 10 min at 37°C.

### 2.5 Total cholesterol measurement

Mice were fasted for 12 h prior to collection of 20 μl blood from the lateral tail vein in a tube containing 2 μl of 0.5 mM EDTA. Plasma total cholesterol levels were measured using Total Cholesterol kit (CH200, Randox) as per manufacturer's instruction.

### 2.6 ELISA for anti‐ds‐DNA and anti‐nucleosome antibodies

High‐binding ELISA plates were coated with poly‐L‐lysine (0.05 mg/ml) for 2 h. The plates were coated with 0.1 mg/ml calf thymus DNA or 1 μg/ml of purified Hela polynucleosomes (Epicypher) overnight for the detection of anti‐ds‐DNA antibodies and anti‐nucleosome antibodies, respectively. After blocking with 3% BSA for 1 h at room temperature, appropriate wells were incubated for 16 h with either human plasma/mouse serum diluted at 1:100 concentration. For calculation of arbitrary units of anti‐dsDNA and anti‐nucleosome antibodies, appropriate wells were incubated with either WHO reference standard for human anti‐dsDNA antibody (15/174, NIBSC, dynamic range of 0–40 U/ml) or pooled sera from lupus‐prone female B6.MRL‐Fas^*lpr*^/J mice containing anti‐nuclear antibodies (dynamic range of 0–25 arbitrary units/mL) in separate ELISA plates and the values were interpolated for measurement of anti‐nuclear antibody levels in test samples. After 3 washes with PBS (phosphate‐buffered saline) containing 0.1% tween‐20, the wells were incubated with 1:1000 dilution of a HRP conjugated anti‐mouse IgG antibody (for mouse samples, Cat# 52‐6520, Invitrogen) or a HRP conjugated anti‐human IgG antibody (for human samples, Cat# A8792, Sigma) for 60 min. After washes, the wells were incubated with TMB (3,3′,5,5′‐Tetramethylbenzidine) substrate for 20 min followed by addition of 2 N H_2_SO_4_ as stop solution. The absorbance was measured at 450 nm. Similarly, anti‐dsDNA IgM and anti‐dsDNA IgG subclasses were measured by ELISA using HRP conjugated antibodies specific to IgM or IgG subclasses (IgM, Cat# 62‐6820, Invitrogen; IgG1 Cat# 1071‐05; IgG2a Cat# 1081‐05; IgG2b Cat# 1091‐05, Southern Biotech).

### 2.7 Immunofluorescence and histopathology

After euthanasia, mice were perfused with PBS via intracardiac puncture and organs were collected in 10% neutral buffered formalin. The tissues were further processed and embedded in paraffin. H&E staining was conducted as described previously.^38^ For IgG deposition in the glomerulus, sections were deparaffinized with histoclear and hydrated using a series of graded ethanol and finally transferred to 1X TBS (Tris‐buffered saline). Antigen retrieval was performed using a heated sodium citrate buffer (10 mM sodium citrate, pH 6.0). For detecting IgG immune complex in kidney, the sections were blocked in 3% FBS in PBS followed by incubation with anti‐mouse IgG conjugated to Alexa flour 647 (Cat# A21236, Invitrogen) for 1 h. After three washes with TBS containing 0.5% tween 20, images were acquired on a fluorescence microscope followed by data analysis in ImageJ. Kidney injury scoring was conducted in H&E and PAS‐stained sections as described previously.^39^, ^40^ Briefly, the analysis included examination of the percentage of tissue demonstrating infiltrating leukocytes, tubular dilatation, cast formation, and loss of tubular brush border with an arbitrary scoring system as follows: 0 = none; 1 = <10%; 2 = 11%–25%; 3 = 26%–45%; 4: 46%–75%; 5 = >75%.

### 2.8 Pico green dye‐based DNase activity assay

For the purpose of high‐throughput and sensitive measurement of DNase activity in human plasma, we optimized a fluorimetry‐based protocol which quantifies the amount of DNA degraded by the enzymatic activity of DNase and converts it into absolute units of DNase activity based on standards of known concentration. Briefly, 1 μg of input DNA was mixed with 1 μl of human plasma or a DNase1 standard of known concentration in a 20 μl reaction buffer containing 20 mM Tris–HCl pH 7.8, 10 mM MnCl_2_, 2 mM CaCl_2_. The assay was conducted for 1 h at 37°C. At the end of reaction, the amount of remaining DNA was quantified using Pico green kit in a NOVOstar fluorimeter (BMG LABTECH) based on manufacturer's protocol.

## 3 RESULTS

### 3.1 Hypercholesterolemia increased the generation of anti‐ds‐DNA and anti‐nucleosome antibodies

**FIGURE 1:**
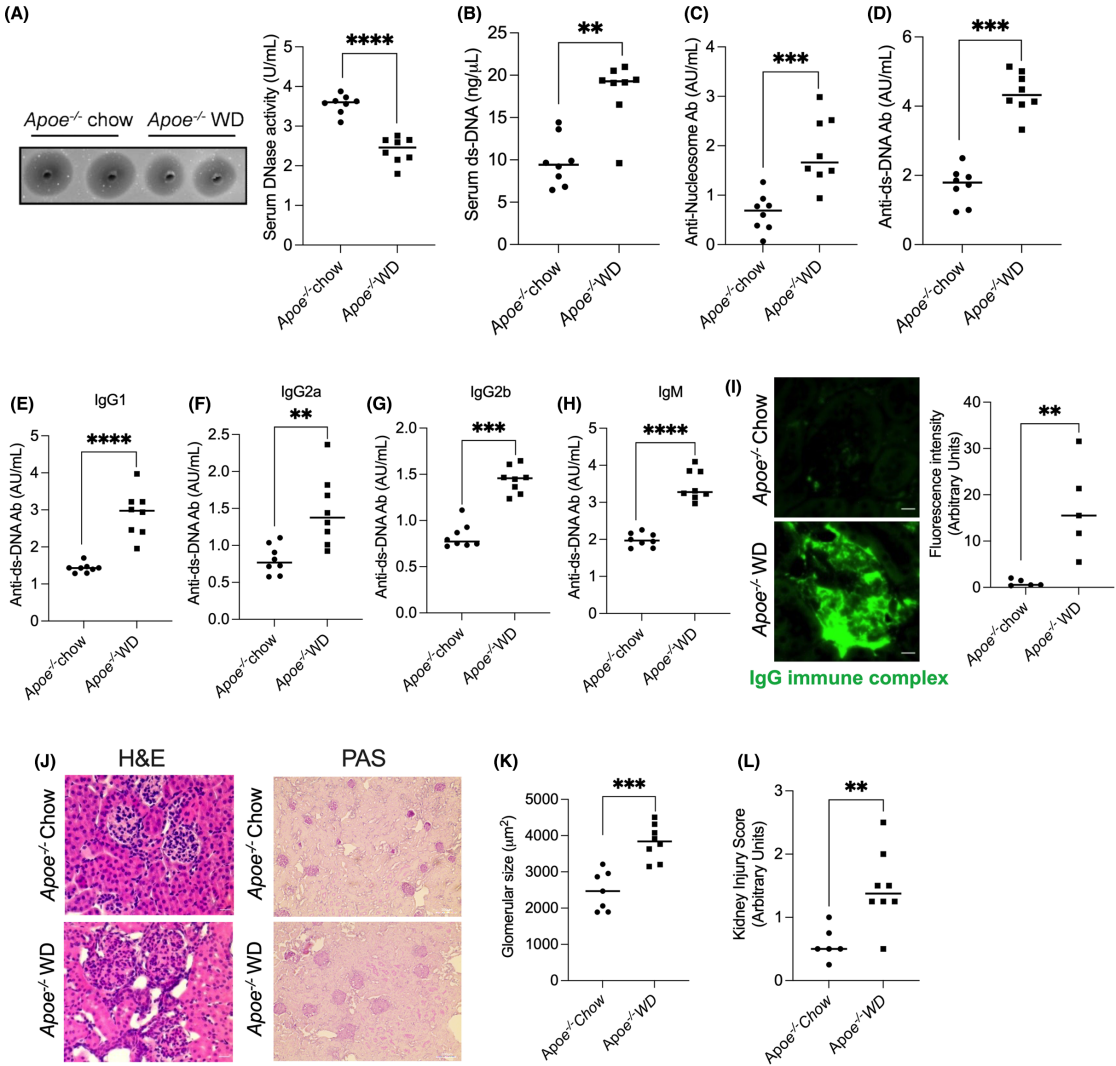
Chronic hypercholesterolemia is associated with increased anti‐nuclear autoantibodies and renal immune complex deposition in *Apoe* ^*−/−*^ mice. *Apoe* ^*−/−*^ mice were fed either a chow diet or a western‐type diet (42% kCal from fat, 0.2% cholesterol) for 16 weeks and the following parameters were analysed. (A) Basal DNase activity in serum was measured using SRED assay. (B) Serum extracellular ds‐DNA level was measured using a Pico green kit. (C and D) ELISA‐based measurement of anti‐ds‐DNA and anti‐nucleosome IgG antibody levels in serum. (E–H) IgM and IgG subclass of anti‐ds‐DNA antibody was measured using ELISA. (I) Sections of kidney were subjected to immunofluorescence analysis for the presence of IgG immune complexes. (J) Kidney sections were stained with H&E (left panel) or PAS (right panel). The size of glomerulus (K) and kidney injury score (L) were analysed. *n* = 8 mice per group. Shapiro–Wilk test was conducted to test for normality of data. Unpaired Student's *t*‐test was conducted to analyse statistical significance between the groups

Hypercholesterolemia is associated with increased levels of anti‐ds‐DNA and anti‐nucleosomal autoantibodies in animal models and humans.^14^, ^35^ We recently demonstrated that hypercholesterolemia in mice is associated with decreased DNase activity and increased systemic ds‐DNA levels.^10^ However, whether the decreased DNase activity and the consequent elevation in systemic ds‐DNA levels is causally linked with enhanced autoantibody responses during hypercholesterolemia is currently unknown. To address this key question, *Apoe* ^*−/−*^ mice were fed either a chow diet or western type diet for 16 weeks to induce hypercholesterolemia. Similar to our observation in short‐term hypercholesterolemic mice,^10^ *Apoe* ^*−/−*^ mice with chronic sustained hypercholesterolemia demonstrated decreased serum DNase activity and increased ds‐DNA levels (Figure 1A,B). Additionally, consistent with previous reports,^34^ we observed a significant increase in the levels of anti‐nucleosome and anti‐ds‐DNA antibody levels in the severely hypercholesterolemic mice (Figure 1C,D). Since the elevation of certain sub‐class of anti‐dsDNA IgG is associated with exacerbated lupus pathology,^41^, ^42^ we next examined the IgG sub‐class distribution of the anti‐ds‐DNA reactive antibodies. Interestingly, the elevation in anti‐DNA antibody level was mediated by an increase in IgM and all IgG subclasses examined including IgG1, IgG2a, and IgG2b (Figure 1E–H). Most importantly, the elevation of autoantibody response in the severely hypercholesterolemic mice was associated with IgG immune complex deposition in the renal glomerulus (Figure 1I) and the associated renal damage manifested as an increase in glomerular size (Figure 1J,K) and an increase in the composite kidney injury score (Figure 1L). These data taken together support the hypothesis that decreased DNase activity enhances autoantibody response and the development of lupus‐like pathology in the hypercholesterolemic mice in part via elevation of antigenic (ds‐DNA) load.

### 3.2 Hypercholesterolemia is associated with increased levels of anti‐ds‐DNA and anti‐nucleosome antibodies in humans

**FIGURE 2:**
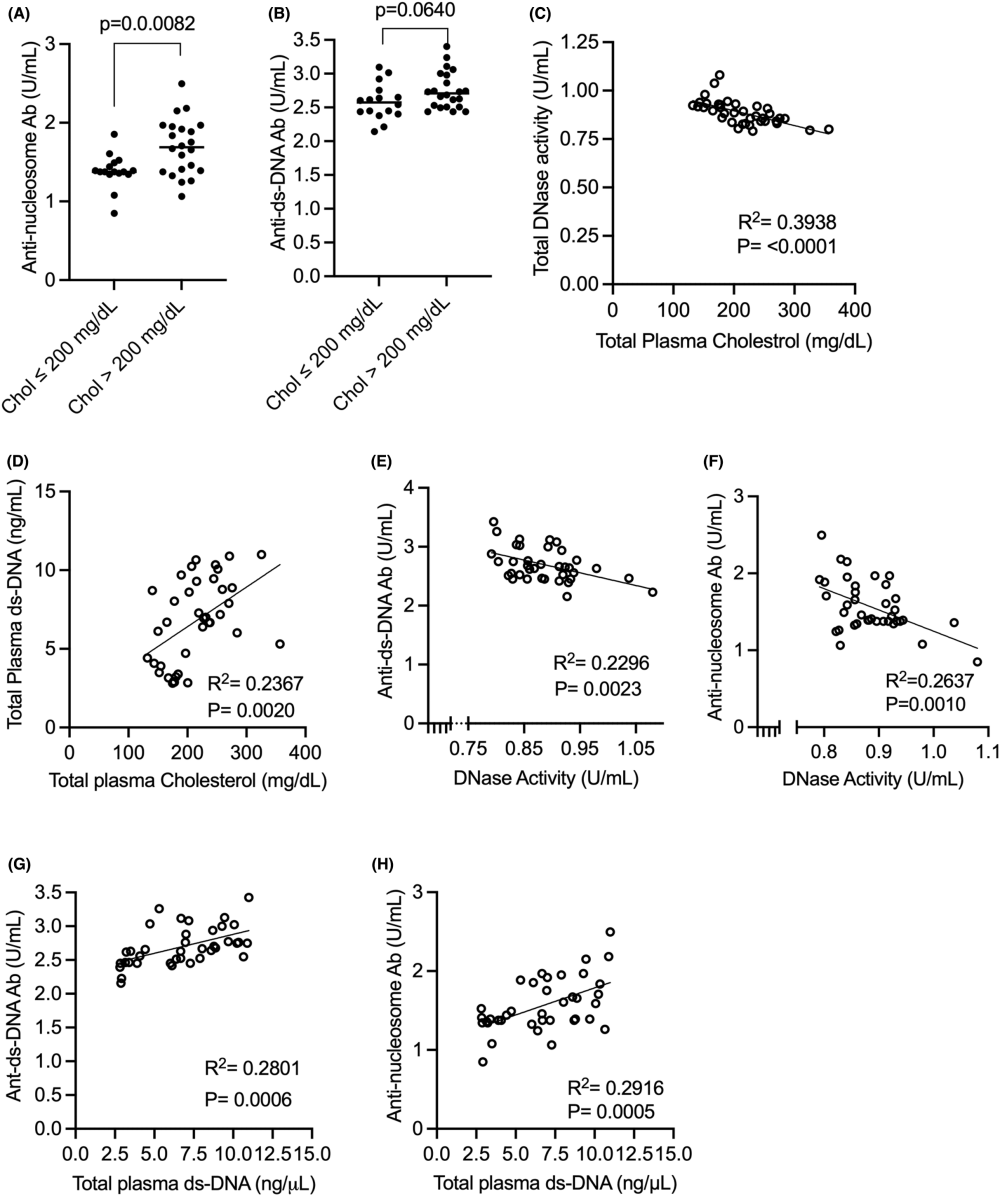
Hypercholesterolemia is associated with decreased DNase activity and increased autoantibody levels in humans. Plasma was collected from healthy adult volunteers and total cholesterol, DNase activity, extracellular ds‐DNA levels, and titres of anti‐ds‐DNA and anti‐nucleosome antibody were quantified. The levels of anti‐nucleosome IgG antibody (A) or anti‐ds‐DNA antibody (B) were compared between individuals with normal cholesterol levels (≤200 mg/dl) and those with hypercholesterolemia (>200 mg/dl). (C) Correlation between DNase activity and total plasma cholesterol. (D) Correlation between plasma ds‐DNA and total plasma cholesterol. (E) Correlation between total DNase activity and anti‐ds‐DNA IgG antibody levels was analysed. (F) Data demonstrates correlation between plasma DNase activity and anti‐nucleosome IgG antibody levels. (G) Correlation analysis between plasma extracellular ds‐DNA and anti‐ds‐DNA IgG antibody levels. (H) Correlation analysis between plasma extracellular ds‐DNA and anti‐nucleosome IgG antibody levels. *n* = 38. Linear regression was conducted to analyse statistical significance

Next, we addressed whether hypercholesterolemia is associated with increased titres of anti‐ds‐DNA and anti‐nucleosome autoantibodies in humans. Towards this end, healthy adults were recruited and their plasma cholesterol, ds‐DNA, DNase activity, and autoantibody levels were quantified. Hypercholesterolemic individuals (plasma total cholesterol >200 mg/dl) demonstrated higher levels of anti‐nucleosome and anti‐ds DNA antibody titres in plasma (Figure 2A,B). As reported previously,^10^ plasma cholesterol level was negatively correlated with DNase activity and positively correlated with plasma extracellular ds‐DNA content in this cohort (Figure 2C,D). Most importantly, DNase activity showed a significant negative correlation with anti‐ds‐DNA/anti‐nucleosome antibody titres (Figure 2E,F) while plasma extracellular ds‐DNA concentration showed a positive correlation with anti‐ds‐DNA/anti‐nucleosome antibody titres (Figure 2G,H). It is important to note that there was no significant difference in the distribution of sex or age between the normocholesterolemic and hypercholesterolemic individuals (50% vs. 45.5% females; 33 ± 7.3 years vs. 34.6 ± 6.8 years, respectively). Since body weight and height were not recorded in the volunteers, alterations in body mass index (BMI) could represent a potential confounding factor. Nevertheless, these data overall raise the possibility that hypercholesterolemia‐induced reduction in systemic DNase activity leads to sustained elevation of extracellular ds‐DNA levels and may be linked to generation of autoantibodies reactive to ds‐DNA and nucleosomes.

### 3.3 Exogenous supplementation of DNase1 lowers autoantibody levels and attenuates lupus‐like pathology

**FIGURE 3:**
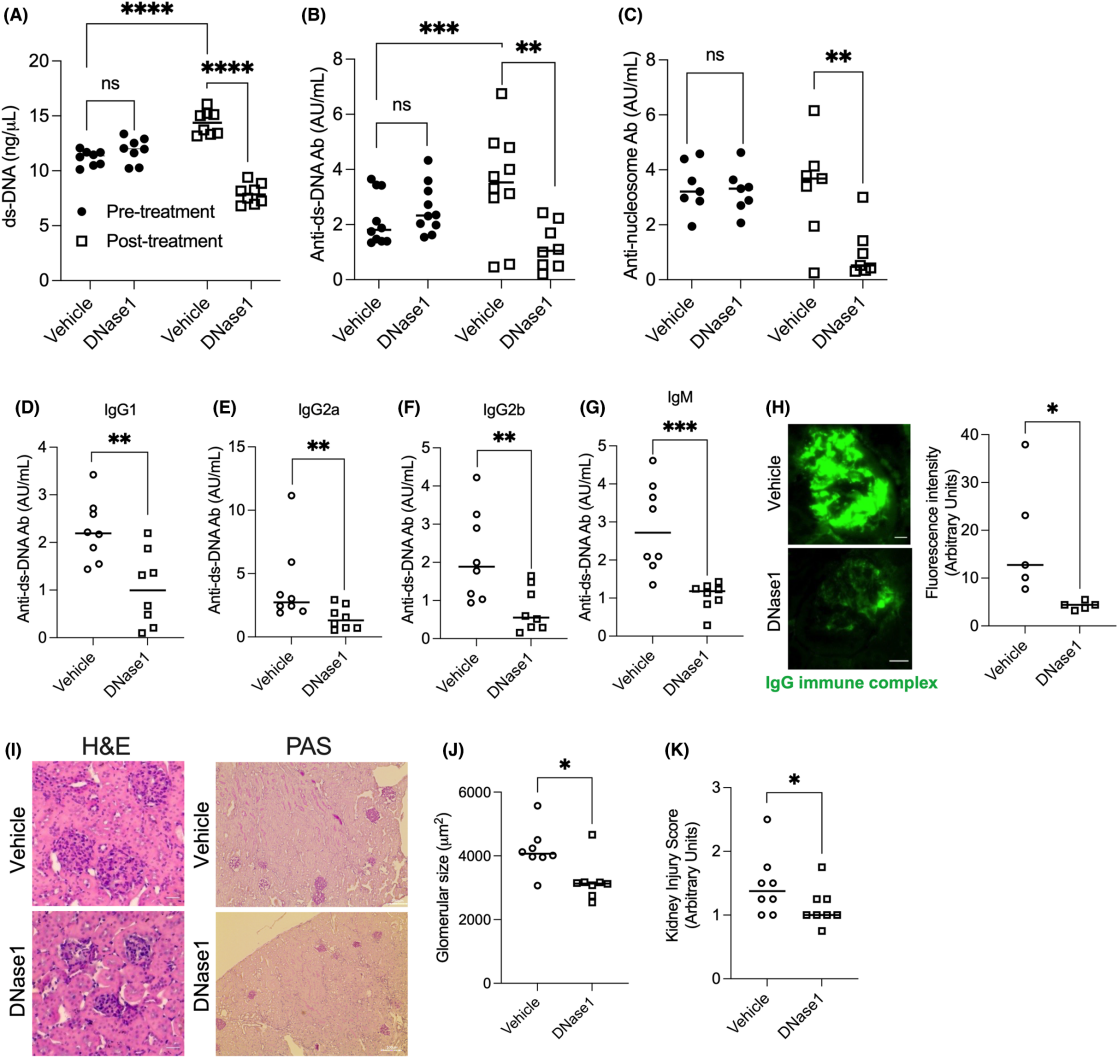
DNase1 treatment attenuates autoantibody levels and renal immune complex deposition in hypercholesterolemic *Apoe* ^*−/−*^ mice. 16‐week WD‐fed *Apoe* ^*−/−*^ mice were administered DNase1 (400 U intraperitoneal, thrice a week) for 4 weeks prior to euthanasia. (A) Extracellular ds‐DNA levels were quantified in the serum of vehicle or DNase1 treated mice. (B) Anti‐ds‐DNA IgG antibody titre or (C) anti‐nucleosome IgG antibody titre was measured in the sera prior to and after injection of vehicle or DNase for 4 weeks. (D–G) Analysis of indicated anti‐ds‐DNA antibody subclasses in the sera of vehicle or DNase1 treated hypercholesterolemic mice. (H) Immunofluorescence analysis of IgG immune complex deposition in kidney sections from vehicle or DNase1‐injected mice. (I) Representative image of kidney sections from vehicle or DNase1‐injected mice stained with H&E (left panel) or PAS (right panel). Glomerular size (J) and kidney injury score (K) were analysed. *n* = 8 mice per group. Statistical significance was calculated by conducting one‐way anova with multiple comparisons test (A–C) or unpaired Student's *t*‐test (D–H, J, K). *p* < 0.05 was considered significant

Next, to directly test the hypothesis that the reduction in DNase activity during hypercholesterolemia is causally linked with increased levels of autoantibodies reactive to DNA and nucleosomes, the systemic levels of DNase in hypercholesterolemic mice was experimentally increased by exogenous supplementation of DNase1. Accordingly, *Apoe* ^*−/−*^ mice were fed western diet for 16 weeks followed by randomization into two groups wherein one group of mice was administered DNase1 (400 U/mouse intraperitoneally three times a week) while the other group received vehicle (PBS) injections for 4 weeks. As predicted, the administration of DNase1 led to a significant decrease in the concentration of plasma extracellular ds‐DNA (Figure 3A). Next, we measured the anti‐ds‐DNA and anti‐nucleosome antibody titres in these two groups of mice prior to and after injection of vehicle or DNase1. The vehicle treated hypercholesterolemic mice showed a significant increase in the levels of anti‐ds‐DNA antibody over the 4‐week period suggesting ongoing immune activation and persistent autoantibody generation (Figure 3B). In contrast and consistent with our hypothesis, the DNase1 treated hypercholesterolemic mice showed a significant reduction in the levels of anti‐ds‐DNA and anti‐nucleosome antibodies (Figure 3B,C). Again, this decrease in autoreactivity was mediated by reduction in the levels of IgM and IgG1, IgG2a, and IgG2b subclass of anti‐ds‐DNA antibodies (Figure 3D–G). Finally, the lowering of systemic autoantibody levels in the DNase1 treated mice was associated with a decrease in IgG immune complex deposition in the renal glomeruli (Figure 3H), improved glomerular morphology (Figure 3I,J), and a decrease in kidney injury score (Figure 3K).

## 4 DISCUSSION

A decrease in DNase activity is strongly correlated with extracellular DNA levels and the progression of several autoimmune disorders including systemic lupus erythematosus and in particular lupus nephritis.^7^, ^11^, ^22^, ^43^ Mechanistically, the decrease in serum DNase activity in patients with lupus and other disorders has been attributed to the presence of DNase activity inhibitors including (a) mutations or SNPs in DNase1/DNase1L3,^15^, ^17^, ^19^, ^20^ (b) anti‐ds‐DNA/anti‐nucleosome antibodies which mask the extracellular DNA and prevent DNase from accessing them,^11^, ^22^ and (c) neutralizing autoantibodies against DNase1 and DNase1L3 which inhibits their enzymatic activity.^21^, ^22^ Given that about 36%–60% of lupus patients develop significant hypercholesterolemia,^26^ our findings suggest a possible fourth mechanism for the decreased serum DNase activity in a subset of these patients, namely, a hypercholesterolemia‐induced absolute decrease in the systemic levels of DNase1 and DNase1L3.

Hypercholesterolemia is known to accelerate the progression of autoimmune pathology in lupus and rheumatoid arthritis.^44^, ^45^, ^46^ Mechanistically, hypercholesterolemia and the consequent lipid accumulation in immune cells leads to a pro‐inflammatory state which is associated with enhanced T‐cell receptor signalling^47^ presumably mediated via abnormal localization of lipid raft‐associated Lck.^48^ Additionally, hypercholesterolemia promotes germinal centre formation and the generation of autoantibodies against ds‐DNA and nucleosome in a TLR4/ IL27‐dependent mechanism.^49^ The findings from our study demonstrate that the exacerbated autoimmune response associated with hypercholesterolemia is mediated in part via an impairment in systemic DNase activity. This decrease in systemic levels of DNase1/DNase1L3 and the consequent perturbation of DNA clearance results in persistent elevation of extracellular DNA in hypercholesterolemic mice and humans.

Extracellular DNA and nucleosomes are highly pro‐inflammatory.^50^ They engage several pattern recognition receptors including TLR7 and TLR9 and the intracellular nucleotide‐sensing systems such as the cGAS‐STING pathway leading to activation of NF‐κB‐mediated pro‐inflammatory signalling.^1^ Consequently, rapid clearance of extracellular DNA via DNase‐mediated enzymatic degradation is a key event to temper pro‐inflammatory responses. Indeed, in a recent study, we demonstrated that there are mechanisms to sense the increase in the level of extracellular DNA which results in rapid release of DNase1 and DNase1L3 from the liver and intestine, respectively, which results in prompt clearance of extracellular DNA and suppression of inflammation.^10^ Since hypercholesterolemia impairs the DNase release response and leads to persistent elevation of extracellular DNA, we speculated that the elevation in antigen load (extracellular DNA) combined with the pro‐immune and pro‐inflammatory environment induced by hypercholesterolemia may account for exacerbated autoimmune responses. Consistent with this hypothesis, our data demonstrated that exogenous supplementation of DNase1 in hypercholesterolemic mice corrected the sub‐optimal DNase release response leading to a decrease in the levels of extracellular DNA and most importantly decreased anti‐ds‐DNA and anti‐nucleosome autoantibody levels. Interestingly, DNase1 treatment in hypercholesterolemic mice also decreased immune complex deposition in the renal glomeruli demonstrating that such a treatment strategy might be beneficial in reversing some of the pathological changes associated with autoimmune nephritis. Whether a similar decrease in autoantibody levels will be observed in normocholesterolemic mice treated with DNase1 remains to be tested. Of note, our previous study^10^ highlighted that normocholesterolemic mice and humans have intact DNA‐induced DNase response. In this context, DNase1 administration in individuals with normal cholesterol levels will represent a pharmacological approach to enhance the clearance of physiological levels of extracellular DNA and the consequences of such an approach are currently unknown.

The occurrence of SLE shows sexual dimorphism with a 9:1 ratio for females vs. males.^51^, ^52^ In this context, our study was conducted exclusively on female mice. However, as shown in our data from humans, both males and females demonstrate an inverse correlation between plasma cholesterol levels and DNase activity suggesting that the phenomenon we report is likely to be applicable to both sexes.

Given the critical role of DNases in several autoimmune disorders,^53^ there is interest around development of DNase‐based therapeutic strategies for alleviation of autoimmune pathology. Indeed, short‐term treatment with recombinant human DNase1 was found to be safe and well tolerated in a clinical trial involving patients with lupus.^54^ No clinical benefit in terms of decrease in autoantibody levels or immune complex deposition was noted during the course of treatment although it is important to note that this study was not sufficiently powered to test treatment efficacy. Since anti‐DNase antibodies are present in a substantial proportion of lupus patients,^21^ recombinant DNase‐based therapies should be adopted in a selectively targeted approach to avoid loss of enzyme efficiency. In this context, our study highlights that hypercholesterolemic individuals without the concomitant presence of anti‐DNase antibody may be good candidates for testing DNase‐based therapies for the alleviation of lupus progression.

In summary, our study demonstrates that decreased DNase activity during hypercholesterolemia and the consequent persistence of extracellular DNA promotes the generation of autoantibodies against ds‐DNA and nucleosomes and the development of a lupus‐like pathology. These data suggest that DNase‐based therapies may be of clinical benefit to select patients who display autoimmune pathology with concomitant hypercholesterolemia.

## AUTHOR CONTRIBUTIONS

**Umesh Kumar Dhawan:** Conceptualization (equal); data curation (equal); formal analysis (equal); investigation (equal); methodology (equal); writing – original draft (equal); writing – review and editing (equal). **Andreas Margraf:** Formal analysis (equal); writing – review and editing (equal). **Maciej Lech:** Methodology (equal); writing – review and editing (equal). **Manikandan Subramanian:** Conceptualization (equal); data curation (equal); formal analysis (equal); funding acquisition (lead); investigation (equal); methodology (equal); project administration (lead); supervision (lead); writing – original draft (equal); writing – review and editing (equal).

## CONFLICT OF INTEREST

The authors declare no conflict of interest.

## Data Availability

The data that support the findings of this study are available from the corresponding author upon request.

## References

[1] Paludan SR , Bowie AG . Immune sensing of DNA. Immunity. 2013;38(5):870‐880. 23706668 10.1016/j.immuni.2013.05.004 PMC3683625

[2] Maronek M , Gromova B , Liptak R , et al. Extracellular DNA correlates with intestinal inflammation in chemically induced colitis in mice. Cells. 2021;10(1). doi:10.3390/cells10010081 PMC7825321 33418977

[3] Truszewska A , Wirkowska A , Gala K , et al. Cell‐free DNA profiling in patients with lupus nephritis. Lupus. 2020;29(13):1759‐1772. 32924831 10.1177/0961203320957717

[4] Lou H , Pickering MC . Extracellular DNA and autoimmune diseases. Cell Mol Immunol. 2018;15(8):746‐755. 29553134 10.1038/cmi.2017.136 PMC6141478

[5] Wu X , Zeng H , Cai L , Chen G . Role of the extracellular traps in central nervous system. Front Immunol. 2021;12:783882. 34868063 10.3389/fimmu.2021.783882 PMC8635093

[6] Wang X , Xia Y . Anti‐double stranded DNA antibodies: origin, pathogenicity, and targeted therapies. Front Immunol. 2019;10:1667. 31379858 10.3389/fimmu.2019.01667 PMC6650533

[7] Duvvuri B , Lood C . Cell‐free DNA as a biomarker in autoimmune rheumatic diseases. Front Immunol. 2019;10:502. 30941136 10.3389/fimmu.2019.00502 PMC6433826

[8] Napirei M , Ludwig S , Mezrhab J , Klockl T , Mannherz HG . Murine serum nucleases‐‐contrasting effects of plasmin and heparin on the activities of DNase1 and DNase1‐like 3 (DNase1l3). FEBS J. 2009;276(4):1059‐1073. 19154352 10.1111/j.1742-4658.2008.06849.x

[9] Napirei M , Wulf S , Eulitz D , Mannherz HG , Kloeckl T . Comparative characterization of rat deoxyribonuclease 1 (Dnase1) and murine deoxyribonuclease 1‐like 3 (Dnase1l3). Biochem J. 2005;389(Pt 2):355‐364. 15796714 10.1042/BJ20042124 PMC1175112

[10] Dhawan UK , Bhattacharya P , Narayanan S , Manickam V , Aggarwal A , Subramanian M . Hypercholesterolemia impairs clearance of neutrophil extracellular traps and promotes inflammation and atherosclerotic plaque progression. Arterioscler Thromb Vasc Biol. 2021;41(10):2598‐2615. 34348488 10.1161/ATVBAHA.120.316389 PMC8454501

[11] Hakkim A , Furnrohr BG , Amann K , et al. Impairment of neutrophil extracellular trap degradation is associated with lupus nephritis. Proc Natl Acad Sci U S A. 2010;107(21):9813‐9818. 20439745 10.1073/pnas.0909927107 PMC2906830

[12] Martinez‐Valle F , Balada E , Ordi‐Ros J , Bujan‐Rivas S , Sellas‐Fernandez A , Vilardell‐Tarres M . DNase 1 activity in patients with systemic lupus erythematosus: relationship with epidemiological, clinical, immunological and therapeutical features. Lupus. 2009;18(5):418‐423. 19318394 10.1177/0961203308098189

[13] Gatselis NK , Vakrakou AG , Zachou K , et al. Decreased serum DNase1‐activity in patients with autoimmune liver diseases. Autoimmunity. 2017;50(2):125‐132. 28263100 10.1080/08916934.2017.1279610

[14] Ryu H , Lim H , Choi G , et al. Atherogenic dyslipidemia promotes autoimmune follicular helper T cell responses via IL‐27. Nat Immunol. 2018;19(6):583‐593. 29713015 10.1038/s41590-018-0102-6

[15] Yasutomo K , Horiuchi T , Kagami S , et al. Mutation of DNASE1 in people with systemic lupus erythematosus. Nat Genet. 2001;28(4):313‐314. 11479590 10.1038/91070

[16] Dittmar M , Bischofs C , Matheis N , Poppe R , Kahaly GJ . A novel mutation in the DNASE1 gene is related with protein instability and decreased enzyme activity in thyroid autoimmunity. J Autoimmun. 2009;32(1):7‐13. 19022625 10.1016/j.jaut.2008.09.005

[17] Zervou MI , Andreou A , Matalliotakis M , Spandidos DA , Goulielmos GN , Eliopoulos EE . Association of the DNASE1L3 rs35677470 polymorphism with systemic lupus erythematosus, rheumatoid arthritis and systemic sclerosis: Structural biological insights. Mol Med Rep. 2020;22(6):4492‐4498. 33173951 10.3892/mmr.2020.11547 PMC7646740

[18] Coke LN , Wen H , Comeau M , et al. Arg206Cys substitution in DNASE1L3 causes a defect in DNASE1L3 protein secretion that confers risk of systemic lupus erythematosus. Ann Rheum Dis. 2021;80:782‐787. 33455918 10.1136/annrheumdis-2020-218810 PMC8142439

[19] Al‐Mayouf SM , Sunker A , Abdwani R , et al. Loss‐of‐function variant in DNASE1L3 causes a familial form of systemic lupus erythematosus. Nat Genet. 2011;43(12):1186‐1188. 22019780 10.1038/ng.975

[20] Bodano A , Gonzalez A , Ferreiros‐Vidal I , et al. Association of a non‐synonymous single‐nucleotide polymorphism of DNASEI with SLE susceptibility. Rheumatology (Oxford). 2006;45(7):819‐823. 16449364 10.1093/rheumatology/kel019

[21] Yeh TM , Chang HC , Liang CC , Wu JJ , Liu MF . Deoxyribonuclease‐inhibitory antibodies in systemic lupus erythematosus. J Biomed Sci. 2003;10(5):544‐551. 12928595 10.1159/000072382

[22] Hartl J , Serpas L , Wang Y , et al. Autoantibody‐mediated impairment of DNASE1L3 activity in sporadic systemic lupus erythematosus. J Exp Med. 2021;218(5):e20201138. 33783474 10.1084/jem.20201138 PMC8020718

[23] Sisirak V , Sally B , D'Agati V , et al. Digestion of chromatin in apoptotic cell microparticles prevents autoimmunity. Cell. 2016;166(1):88‐101. 27293190 10.1016/j.cell.2016.05.034 PMC5030815

[24] Napirei M , Karsunky H , Zevnik B , Stephan H , Mannherz HG , Moroy T . Features of systemic lupus erythematosus in Dnase1‐deficient mice. Nat Genet. 2000;25(2):177‐181. 10835632 10.1038/76032

[25] Kenny EF , Raupach B , Abu Abed U , Brinkmann V , Zychlinsky A . Dnase1‐deficient mice spontaneously develop a systemic lupus erythematosus‐like disease. Eur J Immunol. 2019;49(4):590‐599. 30758851 10.1002/eji.201847875

[26] Tselios K , Koumaras C , Gladman DD , Urowitz MB . Dyslipidemia in systemic lupus erythematosus: just another comorbidity? Semin Arthritis Rheum. 2016;45(5):604‐610. 26711309 10.1016/j.semarthrit.2015.10.010

[27] Reichlin M , Fesmire J , Quintero‐Del‐Rio AI , Wolfson‐Reichlin M . Autoantibodies to lipoprotein lipase and dyslipidemia in systemic lupus erythematosus. Arthritis Rheum. 2002;46(11):2957‐2963. 12428237 10.1002/art.10624

[28] Svenungsson E , Gunnarsson I , Fei GZ , Lundberg IE , Klareskog L , Frostegard J . Elevated triglycerides and low levels of high‐density lipoprotein as markers of disease activity in association with up‐regulation of the tumor necrosis factor alpha/tumor necrosis factor receptor system in systemic lupus erythematosus. Arthritis Rheum. 2003;48(9):2533‐2540. 13130473 10.1002/art.11264

[29] Asanuma Y , Chung CP , Oeser A , et al. Increased concentration of proatherogenic inflammatory cytokines in systemic lupus erythematosus: relationship to cardiovascular risk factors. J Rheumatol. 2006;33(3):539‐545. 16463434

[30] Ajeganova S , Hafstrom I , Frostegard J . Patients with SLE have higher risk of cardiovascular events and mortality in comparison with controls with the same levels of traditional risk factors and intima‐media measures, which is related to accumulated disease damage and antiphospholipid syndrome: a case‐control study over 10 years. Lupus Sci Med. 2021;8(1):e000454. 33547230 10.1136/lupus-2020-000454 PMC7871345

[31] McMahon M , Hahn BH , Skaggs BJ . Systemic lupus erythematosus and cardiovascular disease: prediction and potential for therapeutic intervention. Expert Rev Clin Immunol. 2011;7(2):227‐241. 21426260 10.1586/eci.10.98 PMC3718673

[32] Skaggs BJ , Hahn BH , McMahon M . Accelerated atherosclerosis in patients with SLE—mechanisms and management. Nat Rev Rheumatol. 2012;8(4):214‐223. 22331061 10.1038/nrrheum.2012.14 PMC3765069

[33] Skeoch S , Bruce IN . Atherosclerosis in rheumatoid arthritis: is it all about inflammation? Nat Rev Rheumatol. 2015;11(7):390‐400. 25825281 10.1038/nrrheum.2015.40

[34] Ma Z , Choudhury A , Kang SA , Monestier M , Cohen PL , Eisenberg RA . Accelerated atherosclerosis in ApoE deficient lupus mouse models. Clin Immunol. 2008;127(2):168‐175. 18325838 10.1016/j.clim.2008.01.002 PMC2464279

[35] Hutchinson MA , Park HS , Zanotti KJ , et al. Auto‐antibody production during experimental atherosclerosis in ApoE(−/−) mice. Front Immunol. 2021;12:695220. 34305930 10.3389/fimmu.2021.695220 PMC8299997

[36] Wang Y , Huang Z , Lu H , et al. Apolipoprotein E‐knockout mice show increased titers of serum anti‐nuclear and anti‐dsDNA antibodies. Biochem Biophys Res Commun. 2012;423(4):805‐812. 22713470 10.1016/j.bbrc.2012.06.044

[37] Lewis MJ , Malik TH , Fossati‐Jimack L , et al. Distinct roles for complement in glomerulonephritis and atherosclerosis revealed in mice with a combination of lupus and hyperlipidemia. Arthritis Rheum. 2012;64(8):2707‐2718. 22392450 10.1002/art.34451 PMC3607248

[38] Fischer AH , Jacobson KA , Rose J , Zeller R . Hematoxylin and eosin staining of tissue and cell sections. Cold Spring Harb Protoc. 2008;2008:pdb prot4986. 10.1101/pdb.prot4986 21356829

[39] Kuriakose J , Redecke V , Guy C , et al. Patrolling monocytes promote the pathogenesis of early lupus‐like glomerulonephritis. J Clin Invest. 2019;129(6):2251‐2265. 31033479 10.1172/JCI125116 PMC6546471

[40] Li L , Huang L , Vergis AL , et al. IL‐17 produced by neutrophils regulates IFN‐gamma‐mediated neutrophil migration in mouse kidney ischemia‐reperfusion injury. J Clin Invest. 2010;120(1):331‐342. 20038794 10.1172/JCI38702 PMC2798679

[41] Bijl M , Dijstelbloem HM , Oost WW , et al. IgG subclass distribution of autoantibodies differs between renal and extra‐renal relapses in patients with systemic lupus erythematosus. Rheumatology (Oxford). 2002;41(1):62‐67. 11792881 10.1093/rheumatology/41.1.62

[42] Lin GG , Li JM . IgG subclass serum levels in systemic lupus erythematosus patients. Clin Rheumatol. 2009;28(11):1315‐1318. 19597698 10.1007/s10067-009-1224-x

[43] Sallai K , Nagy E , Derfalvy B , Muzes G , Gergely P . Antinucleosome antibodies and decreased deoxyribonuclease activity in sera of patients with systemic lupus erythematosus. Clin Diagn Lab Immunol. 2005;12(1):56‐59. 15642985 10.1128/CDLI.12.1.56-59.2005 PMC540196

[44] Prieto‐Potin I , Roman‐Blas JA , Martinez‐Calatrava MJ , Gomez R , Largo R , Herrero‐Beaumont G . Hypercholesterolemia boosts joint destruction in chronic arthritis. An experimental model aggravated by foam macrophage infiltration. Arthritis Res Ther. 2013;15(4):R81. 23941259 10.1186/ar4261 PMC3978700

[45] Turesson C , Bergstrom U , Pikwer M , Nilsson JA , Jacobsson LT . High serum cholesterol predicts rheumatoid arthritis in women, but not in men: a prospective study. Arthritis Res Ther. 2015;17:284. 26458977 10.1186/s13075-015-0804-1 PMC4603637

[46] Handel ML , Simons L . Rheumatic manifestations of hyperlipidaemia. Baillieres Best Pract Res Clin Rheumatol. 2000;14(3):595‐598. 10985988 10.1053/berh.2000.0095

[47] Proto JD , Doran AC , Subramanian M , et al. Hypercholesterolemia induces T cell expansion in humanized immune mice. J Clin Invest. 2018;128(6):2370‐2375. 29708512 10.1172/JCI97785 PMC5983331

[48] Jury EC , Isenberg DA , Mauri C , Ehrenstein MR . Atorvastatin restores Lck expression and lipid raft‐associated signaling in T cells from patients with systemic lupus erythematosus. J Immunol. 2006;177(10):7416‐7422. 17082661 10.4049/jimmunol.177.10.7416

[49] Ryu H , Chung Y . Dyslipidemia promotes germinal center reactions via IL‐27. BMB Rep. 2018;51(8):371‐372. 30037367 10.5483/BMBRep.2018.51.8.171 PMC6130833

[50] Paludan SR . Activation and regulation of DNA‐driven immune responses. Microbiol Mol Biol Rev. 2015;79(2):225‐241. 25926682 10.1128/MMBR.00061-14 PMC4429241

[51] Petri M . Epidemiology of systemic lupus erythematosus. Best Pract Res Clin Rheumatol. 2002;16(5):847‐858. 12473278 10.1053/berh.2002.0259

[52] Weckerle CE , Niewold TB . The unexplained female predominance of systemic lupus erythematosus: clues from genetic and cytokine studies. Clin Rev Allergy Immunol. 2011;40(1):42‐49. 20063186 10.1007/s12016-009-8192-4 PMC2891868

[53] Keyel PA . Dnases in health and disease. Dev Biol. 2017;429(1):1‐11. 28666955 10.1016/j.ydbio.2017.06.028 PMC11492367

[54] Davis JC Jr , Manzi S , Yarboro C , et al. Recombinant human Dnase I (rhDNase) in patients with lupus nephritis. Lupus. 1999;8(1):68‐76. 10025601 10.1191/096120399678847380

